# The A Allele of the rs1990760 Polymorphism in the *IFIH1* Gene Is Associated with Protection for Arterial Hypertension in Type 1 Diabetic Patients and with Expression of This Gene in Human Mononuclear Cells

**DOI:** 10.1371/journal.pone.0083451

**Published:** 2013-12-27

**Authors:** Ana P. Bouças, Letícia A. Brondani, Bianca M. Souza, Natália E. Lemos, Fernanda S. de Oliveira, Luis H. Canani, Daisy Crispim

**Affiliations:** 1 Laboratory of Biology of Human Pancreatic Islet, Endocrinology Division, Hospital de Clínicas de Porto Alegre, Universidade Federal do Rio Grande do Sul, Porto Alegre, RS, Brazil; 2 Postgraduate Program in Medical Sciences, Endocrinology, Universidade Federal do Rio Grande do Sul, Porto Alegre, RS, Brazil; Sudbury Regional Hospital, Canada

## Abstract

**Background:**

The rs1990760 polymorphism of interferon induced with helicase C domain 1 (*IFIH1*) has been associated with type 1 diabetes mellitus (T1DM). Here, we investigated whether this polymorphism is associated with T1DM or its clinical characteristics in a Brazilian population, and if *IFIH1* gene expression in mononuclear cells from T1DM patients differs according to the genotypes of this polymorphism. A meta-analysis was also conducted to evaluate if the rs1990760 polymorphism is associated with T1DM.

**Methods:**

Frequencies of the rs1990760 polymorphism were analyzed in 527 T1DM patients and in 517 healthy subjects. *IFIH1* gene expressions according to genotypes were measured in a sub-sample of 26 T1DM patients by quantitative real-time PCR.

**Results:**

Our data show the association of the A allele with risk to T1DM under a dominant model of inheritance [odds ratio (OR) = 1.421, P = 0.037], adjusting for ethnicity. The meta-analysis revealed significant association between the rs199760A allele and risk for T1DM for all analyzed inheritance models. Surprisingly, T1DM patients carrying the A allele showed lower levels of systolic (P = 0.001) and diastolic (P = 1×10^−10^) blood pressures as compared to G/G carriers. Furthermore, the A/A genotype seems to be associated with protection to arterial hypertension (AH) after adjustment for covariates (OR = 0.339, P = 0.019). *IFIH1* gene expression in mononuclear cells from 26 T1DM patients did not differ among genotypes (P = 0.274). Nevertheless, *IFIH1* gene expression was increased in mononuclear cells from T1DM patients with AH as compared with T1DM patients without AH [6.7 (1.7–2.0) *vs*. 1.8 (1.3–7.1) arbitrary units; P = 0.036]. The association with blood pressures and AH was not observed in patients with type 2 diabetes mellitus.

**Conclusions:**

Our results indicate that the rs1990760 polymorphism is associated with T1DM. Interestingly, the rs1990760 A allele seems to be associated with protection for AH in T1DM patients. Further studies are needed to confirm the association with AH.

## Introduction

Type 1 diabetes mellitus (T1DM), which accounts for 5–10% of those with diabetes, results from a cellular-mediated autoimmune destruction of the pancreatic beta-cells, which renders patients insulin-dependent for life [1]. The triggering of autoimmunity against beta-cells is probably caused by environmental factors acting in combination with a predisposing genetic background [2], [3]. The major susceptibility locus maps to the HLA class II genes at chromosome 6p21 and accounts for up to 30–50% of the genetic risk for this disease [1], [4]. Other non-HLA loci have smaller effects on disease risk compared to HLA, and include the *insulin* gene, the *CTLA4* gene, the *PTPN22* gene, the *IL2RA* gene, and the interferon induced with helicase C domain 1 (*IFIH1*) gene [4].

The hypothesis that viruses may be one of the environmental factors explaining the surprising increase in T1DM incidence during recent decades is strengthened by variations of T1DM incidence from one country to another and from a season to another, as well as by observations relative to the association between immigration and disease development [5]. Epidemiological, experimental and clinical data indicate that the prime viral candidates for causing T1DM in human are enteroviruses, such as Coxsackievirus B (CV-B) [5], [6], [7]. CV-B4 is the most common enterovirus found in pre-diabetic and diabetic subjects [7]. One CV-B4 strain isolated from the pancreas of a deceased diabetic child was able to induce diabetes in susceptible mice [8]. Moreover, CV-B4 was identified in the pancreatic tissue from three of six patients with T1DM [9], and it was capable of infecting human islet *in vitro*, impairing glucose-stimulated insulin secretion [10]. The pathogenic role of enteroviruses in T1DM seems to involve damage to beta-cells and local induction of proinflammatory mediators [11].

The immune response to virus infection begins with the recognition of pathogen-associated molecular patterns (PAMPs) as “nonself” signatures. This recognition occurs through host pattern recognition receptors (PRRs), and triggers intracellular signaling events that induce innate immunity, the front line of defense against microbial infection. PRRs are evolutionary conserved germ-line-encoded proteins and include Toll-like receptors (TLRs), retinoic acid-inducible I (RIG-I)-like receptors (RIG-I and IFIH1 receptors) and nucleotide-binding oligomerization domain-like receptor (NLR) [12]. These PRRs recognize specific PAMPs in different cellular compartments, such as the plasma membrane, the endossomes or the cytoplasm, and induce the expression of proinflammatory cytokines, chemokines and co-stimulatory molecules which will eliminate the pathogens and activate pathogen-specific adaptive immune responses [12], [13].

The *IFIH1* gene, also known as melanome differentiation-associated gene 5 (*MDA5*), is a functional candidate for T1DM because it encodes a cytosolic receptor that play a major role in the recognition of internal double-stranded RNA (dsRNA), an intermediate nucleic acid generated during the life cycle of most viruses [12], [14], suggesting a potential role in the infectious etiology of T1DM and providing a link between viral infections and this disease. In fact, some studies have demonstrated the association of a non-synonymous polymorphism [rs1990760 G/A (Ala946Thr)] in exon 15 of the *IFIH1* gene with T1DM in more than one population [15], [16], [17]. Therefore, the present study investigated whether the *IFIH1* rs1990760 polymorphism is associated with T1DM or its clinical and laboratory characteristics in a Southeast Brazilian population, and if the *IFIH1* gene expression in mononuclear cells from T1DM patients differs according to the different genotypes of this polymorphism. Moreover, a meta-analysis was conducted to attempt determining if the *IFIH1* rs1990760 polymorphism is associated with T1DM.

## Methods

### Case-control Study and Analysis of the *IFIH1* Gene Expression

#### Ethical approval of the research protocol

The information obtained from the study did not influence patients’ diagnosis or treatment. The study protocol was approved by Ethic Committee in Research from Hospital de Clínicas de Porto Alegre and all patients and nondiabetic subjects provided informed consent in writing. All clinical investigation has been conducted according to the principles expressed in the Declaration of Helsinki.

#### Subjects and phenotype measurements

This was a case-control study designed to investigate whether the *IFIH1* rs1990760 polymorphism is associated with T1DM. The diabetic sample comprised 527 unrelated patients from the outpatient clinic at the Hospital de Clínicas de Porto Alegre (Rio Grande do Sul, Brazil). Patients were considered to have T1DM if they had been diagnosed with hyperglycemia before the age of 30 years, required insulin for glycemic control within 1 year of diagnosis, and this treatment could not be interrupted thereafter. The nondiabetic sample comprised 517 healthy blood donors who did not have diabetes mellitus or family history for this disease (mean age = 44.0±7.8; male = 55.0%). Additionally, to evaluate whether any association found between clinical characteristics of T1DM and the *IFIH1* rs1990760 polymorphism could be replicated in a non-autoimmune diabetes context, we also analyzed 725 patients with type 2 diabetes mellitus (T2DM) from the same hospital [18]. T2DM was diagnosed according to the ADA guidelines [1], and clinical and laboratory characteristics of these patients can be found in **Table S1 in File S1**. The ethnic group was defined on the basis of self-classification, and the ethnic proportion was similar between the samples: 16.1% of black in T1DM group and 19.8% of black subjects in the nondiabetic group (P = 0.110).

A standard questionnaire was used to collect information on age, age at DM diagnosis, and drug treatment and all patients underwent physical and laboratory evaluations. They were weighed unshod, wearing light outdoor clothes and their height was measured. Body mass index (BMI) was calculated as weight (kg)/height square (meters). Blood pressure (BP) was measured by a trained researcher, with a mercury sphygmomanometer on the left arm, using an appropriated cuff size, in a sitting position, after a 5-min rest. The mean of two measurements taken 1 min apart was used to calculate systolic and diastolic BP. Arterial hypertension (AH) was defined as BP levels higher than 140/90 mmHg at initial visit and at two follow-up visits within 1 month of the initial visit, or if the presence of AH was registered on medical records. Assessment of diabetic retinopathy (DR) was performed in all patients by an experienced ophthalmologist using fundoscopy through dilated pupils. DR was classified using the scale developed by the Global Diabetic Retinopathy Group [19]. For the purpose of this study, patients were grouped according to the presence or absence of any degree of DR. Diagnosis of diabetic nephropathy (DN) was based on the albumin excretion rate (AER) in at least two out of three consecutive 24-h timed or random spot sterile urine collections in a 6-month period. Patients were classified as having normoalbuminuria (AER <30 µg/24 h or <17 mg/l), microalbuminuria (AER 30–299 µg/24 h or 17–173 mg/l) or macroalbuminuria (AER >300 µg/24 h or >174 mg/l) [20].

Serum and plasma samples were taken after a 12 hours of fasting for laboratory analyses. Plasma glucose levels were determined using the glucose oxidase method. Creatinine levels were determined using the Jaffe reaction. Glycated hemoglobin (HbA1c) measurements were performed by different methods and results were traceable to the DCCT method by off-line calibration or through conversion formulae [21]. Total plasma cholesterol, HDL cholesterol and triglycerides were assayed using enzymatic methods. Urinary albumin was measured by immunoturbidimetry (Microalb; Ames-Bayer, Tarrytown NY), and the intra- and interassay coefficients of variation in our laboratory were both <6% [22].

#### Genotyping

DNA was extracted from peripheral blood leukocytes using a standardized salting-out procedure. The *IFIH1* rs1990760 (G/A) polymorphism was genotyped using primers and probes contained in the Human Custom TaqMan Genotyping Assay 20× (Assays-By-Design Service; Life Technologies, Foster City, CA). Sequences of primers and probes were: 5′-ACCATTTATTTGATAGTCGGCACACT-3′ (forward); 5′ CTCCATGATGATTCTTTCCCTTTGATACTT-3′ (reverse); 5′-AAGAGAAAACAAAGCACTGC-3′ (VIC; specific to the G allele) and 5′-AAGAGAAAACAAAACACTGC-3′ (FAM, specific to the A allele). Reactions were conducted in 96-well plates, in a total 5 µl volume using 2 ng of genomic DNA, TaqMan Genotyping Master Mix 1× (Life Technologies) and Custom TaqMan Genotyping Assay 1×. The plates were then positioned in a thermal cycler (7500 Fast Real-Time PCR System; Life Technologies) and heated for 10 min at 95°C, followed by 50 cycles of 95°C for 15 s and 63°C for 1 min. The genotyping success rate was better than 95%, with a calculated error rate based on PCR duplicates of less than 1%.

#### Isolation of mononuclear cells from peripheral blood of patients with T1DM and RNA extraction

Samples of 10 ml peripheral blood were collected from a sub-sample of 26 T1DM patients belonging to our case-control study. Immediately after collecting the samples, an aliquot of 2 ml of blood was mixed with an equal volume of PBS. Then, total mononuclear cells were isolated from blood by density gradient centrifugation using Ficoll-paqueTM plus reagent (GE HealthCare, Uppsala, Sweden). Isolated mononuclear cells were stored at −80°C until RNA extractions.

Total RNA was extracted from mononuclear cells using the RNeasy Mini kit (Qiagen, Hilden, Germany). The concentration and quality of total RNA samples were assessed using a NANODROP 2000 spectrophotometer (Thermo Scientific Inc., Newark, DE). Only RNA samples with adequate purity ratios (A260/A280 = 1.9–2.1) were used for subsequent analyses [23]. In addition RNA integrity and purity were also checked on agarose gel containing GelRed Nucleic Acid Gel Stain (Biotium Inc., Hayward, CA).

#### Quantification of *IFIH1* gene expression by Real-Time PCR

Real-Time PCR was performed in two separate reactions: first, RNA was reverse transcribed into cDNA, then cDNA was amplified by quantitative real-time PCR (RT-qPCR). Reverse transcription of 1 µg of RNA into cDNA was carried out using the High Capacity cDNA Reverse Transcription Kit (Life Technologies), following the manufacturer’s protocol for oligo (dT) method.

RT-qPCR experiments were performed in a Vii™ 7 Real-Time PCR System (Life Technologies). Experiments were performed by real-time monitoring of the increase in fluorescence of SYBER® Green dye [24]. Primers for the target (*IFIH1*) and reference (*GAPDH)* genes were: *IFIH1* 5′- ATGGAAAAAAAAGCTGCAAAAGA -3′ (forward), *IFIH1* 5′-GTACTTCCTCAAATGTTCTGCACAA -3′ (reverse), *GAPDH* 5′- ACCCACTCCTCCACCTTTG –3′ (forward) and *GAPDH* 5′- CTCTTGTGCTCTTGCTGGG –3′ (reverse). PCR reactions were performed using 5 µl of 2x Fast SYBER® Green Master Mix (Life Technologies), 0.5 µl (0.5 ng/µl) of forward and reverse primers for *IFIH1* or *GAPDH*, and 0.5 µl of cDNA template (1 µg/µl), in a total volume of 10 µl. Each sample was assayed in triplicate and a negative control was included in each experiment. The thermocycling conditions for these genes were as follows: an initial cycle of 95°C for 20 s, followed by 50 cycles of 95°C for 5 s and 60°C for 1 min. RT-qPCR specificity was determined using melting curve analyses and all primers generated amplicons that produced a single sharp peak during the analyses.

Quantification of the *IFIH1* mRNA was performed using the relative standard curve method [23], [25], and the *GAPDH* gene as the reference gene. Relative standard curves were generated for both target and reference genes by preparing serial dilutions of the same cDNA sample with a known relative quantity. Then, relative amounts of each *IFIH1* cDNA sample were obtained by normalizing their signals by those of *GAPDH* gene, and are presented as arbitrary units (AU).

#### Statistical analyses

Allelic frequencies were determined by gene counting, and departures from the Hardy–Weinberg equilibrium (HWE) were verified using the χ^2^-test. Allele and genotype frequencies were compared between groups of patients using χ^2^-tests. Clinical and laboratory characteristics and mRNA abundance were compared between groups by using unpaired Student’s t-test, One-way ANOVA or χ^2^, as appropriate. Variables with normal distribution are presented as mean ± SD or percentage. Variables with skewed distribution were log-transformed before analyses and are presented as median (minimum-maximum values). The magnitude of associations between rs1990760 genotypes and T1DM or its categorical clinical characteristics were estimated using OR (95% CI). Multivariate logistic regression analyses were performed to assess the independent association of the rs1990760 polymorphisms with T1DM or its categorical clinical characteristics and to control for possible confounding factors whenever a statistically significant association was detected by univariate analyses.

Results for which P was less than 0.05 were considered statistically significant. Bonferroni correction was used to account for multiple comparisons. These statistical analyses were performed using SPSS version 18.0 (SPSS, Chicago, IL).

Power calculations (PEPI program, version 4.0) showed that this study has a power of approximately 80% at a significance level of 0.05 to detect an odds ratio of 1.45 (for the presence of the A allele). However, our power is less than 80% when considering lower ORs.

### Meta-analysis

#### Search strategy and eligibility criteria

**Figure 1:**
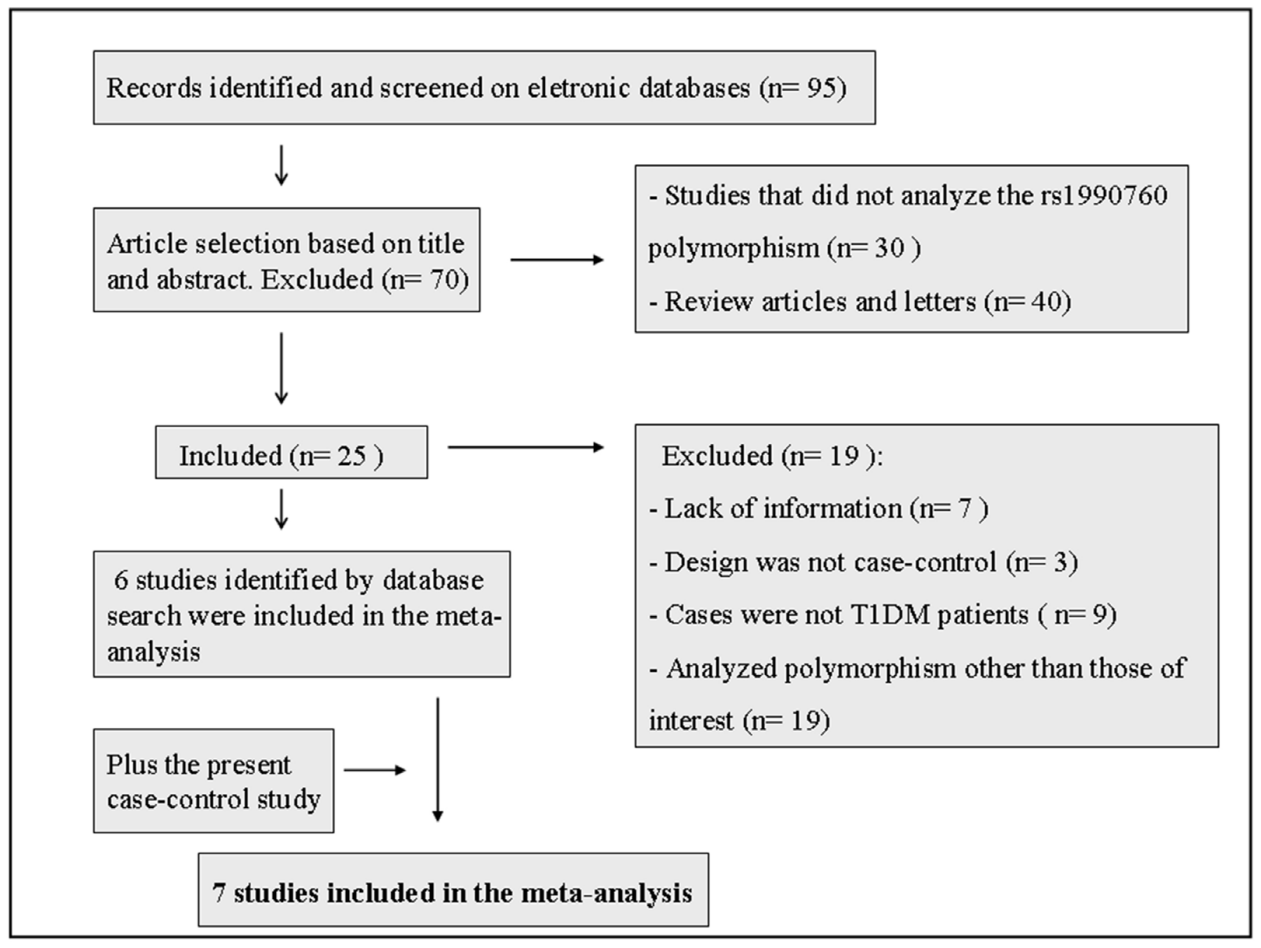
Flowchart illustrating the search strategy used to identify association studies of the *IFIH1* rs1990760 polymorphism and type 1 diabetes mellitus for the meta-analysis. The following medical subject headings (MESH) were used for searching in the electronic databases: (“Diabetes Mellitus, Type 1” OR “Autoimmune Diseases”) AND (“Polymorphism, Genetic” OR “Polymorphism, Single Nucleotide” OR “Polymorphism, Restriction Fragment Length” OR “DNA Copy Number Variations”) AND (Search “Mutation” OR “INDEL Mutation” OR “Mutation, Missense” OR “Germ-Line Mutation” OR “Point Mutation” OR “Frameshift Mutation” OR “Codon, Nonsense” OR “Sequence Deletion”) AND (“MDA-5 OR IFIH1 protein, human”).

This study was designed and described in accordance with current guidelines [26], [27]. PubMed and Embase were searched to identify all genetic studies of associations between T1DM and the *IFIH1* rs1990760 polymorphism. The medical subject headings (MeSH) used for this search are shown in **Figure 1**. The search was completed on September 10, 2013. Two investigators (F.S.O and A.P.B.) independently reviewed the titles and abstracts of all articles selected in order to evaluate whether the studies were eligible for inclusion in the meta-analysis. Where abstracts did not provide enough information regarding the inclusion and exclusion criteria, the full text of the article was retrieved for evaluation. We included observation studies (case-control or cross-sectional designs) that compared the rs1990760 polymorphism between a known number of T1DM patients and nondiabetic subjects. Studies were excluded from the analysis if the genotype distributions in control group deviated from those predicted by the HWE or if they did not have sufficient data to estimate an OR with 95% CI. Data were independently extracted by two investigators (A.P.B and B.M.S) using a standardized abstraction form, and consensus was sought in all extracted items. When consensus could not be reached, differences in data extraction were resolved by a third reviewer (D.C). Two investigators (A.P.B and B.M.S) independently assessed the quality of each eligible study using the Newcastle-Ottawa Scale (NOS) for assessing quality of case-control studies in meta-analysis [28]. The NOS scale contains eight items, categorized into three dimensions including selection, comparability, and exposure. For each item a series of response options is provided. A star system is used to allow a semi-quantitative assessment of the study quality, and the total NOS score ranges from zero to nine stars.

#### Statistical analyses for meta-analysis

Control subjects’ genotype distributions were tested for conformity with HWE using a goodness-of-fitness χ^2^ test. Gene-disease associations were measured using OR (95% CI) estimation based on the following genetic inheritance models: (1) allele contrast; (2) additive model; (3) recessive model and (4) dominant model (5) [29], [30]. Heterogeneity was tested using a χ^2^-based Cochran’s Q statistic and inconsistency was tested using the I^2^ metric. Heterogeneity was considered statistically significant at P<0.10 for the Q statistic and I^2^>50% [31], [32]. Since no significant heterogeneity was found, the fixed effect model (FEM) was used to calculate OR (95% CI) for each individual study and for the pooled effect. Risk of publication bias was assessed using funnel plot graphics, analyzed both visually and with the Begg and Egger test [33]. P values <0.10 were considered indicative of statistically significant publication bias. All statistical analyses were performed using Stata 11.0 software (StataCorp, College Station, TX, USA).

## Results

### Sample Description

The main clinical and laboratory characteristics of the 527 T1DM patients belonging to the present study were as follows: mean age was 33.6±11.8 years; mean age at T1DM diagnosis was 17.4±10.2 years; mean HbA1c was 8.7±4.1%; and mean BMI was 24.7±4.3 kg/m^2^. Males comprised 48.0% of the sample, 16.1% were black, 28% of all patients had AH, 45.1% had some degree of DR, and 35.2% had some degree of DN.

### Study of the Association between the *IFIH1* rs1990760 (G/A) Polymorphism and Type 1 Diabetes Mellitus or its Clinical and Laboratory Characteristics

Genotype and allele frequencies of the rs1990760 (G/A) polymorphism in T1DM patients and nondiabetic subjects are depicted in **Table 1** **.** The frequency of the A allele was 29.4% in white subjects and 6.3% in black subjects (P<0.001). Neither genotype nor allele frequencies of the rs1990760 polymorphism differed statistically between T1DM patients and nondiabetic subjects (P = 0.139 and P = 0.129, respectively), and all genotypes were in agreement with those predicted by the HWE in the two samples (P>0.05). After adjustment for ethnicity, the A allele showed only a trend towards association with T1DM (A/G genotype: OR = 1.403, P = 0.058; A/A genotype: OR = 1.453, P = 0.061). However, the presence of the A allele was significantly associated with risk to T1DM under a dominant model of inheritance, adjusting for ethnicity (OR = 1.421, P = 0.037) (**Table 1**). Of note, after the exclusion of black patients from the sample, the presence of the A allele was significantly associated with risk to T1DM in white subjects under a dominant model of inheritance (OR = 1.381, 95% CI 1.003–1.903; P = 0.048).

**Table 1:** Genotype and allele frequencies of the *IFIH1* rs1990760 G/A polymorphism in patients with type 1 diabetes mellitus (T1DM) and nondiabetic subjects.

	T1DM patients (n = 527)	Nondiabetic subjects (n = 517)	Unadjusted P*	Adjusted OR (95% CI)/P†
Genotype				
G/G	114 (21.6%)	139 (26.9%)	0.139	1
G/A	263 (49.9%)	239 (46.2%)		1.403 (0.989–1.991)/0.058
A/A	150 (28.5%)	139 (26.9%)		1.453 (0.983–2.147)/0.061
Allele				
G	0.466	0.500	0.129	
A	0.534	0.500		
Additive model				
G/G	114 (43.2%)	139 (50.0%)	0.133	1
A/A	150 (56.8%)	139 (50.0%)		1.471 (0.989–2.187)/0.056
Recessive model				
G/A-G/G	377 (71.5%)	378 (73.1%)	0.617	1
A/A	150 (28.5%)	139 (26.9%)		1.157 (0.850–1.575)/0.354
Dominant model				
G/G	114 (21.6%)	139 (26.9%)	0.048	1
G/A-A/A	413 (78.4%)	378 (73.1%)		1.421 (1.022–1.975)/0.037

Data are presented as number (%) or proportion.

P values were computed by χ^2^ tests comparing T1DM patients and nondiabetic subjects.

Adjusted OR (95% CI) and P values obtained by logistic regression analysis controlling for ethnicity.

In order to determine if the rs1990760 polymorphism influenced the severity of T1DM, we re-analyzed the data only including those patients with early-onset of T1DM (age at diagnosis <17 years) [16]. The frequency of the A allele also was increased in early-onset T1DM patients as compared to nondiabetic subjects, although this comparison did not reach formal statistical significance (82.3% *vs*. 76.5%, respectively; P = 0.064 for the dominant model), probably due to the smaller sample size.

Clinical and laboratory characteristics of T1DM patients broken down by the different genotypes of the rs1990760 polymorphism are shown in **Table 2**. Age, age at T1DM diagnosis, proportion of males, BMI, creatinine levels, serum total cholesterol, triglycerides, HbA1c and presence of DR or DN did not differ statistically among the three genotypes. Interestingly, T1DM patients carrying the A allele showed lower levels of systolic BP as compared to patients homozygous for the G allele (A/A: 117.4±16.7, A/G: 121.1±19.0, G/G; 128.5±18.9 mmHg; P = 0.001), taking into consideration a Bonferroni threshold of 0.0038. After Bonferroni correction, patients carrying the A/A or G/A genotypes also had lower levels of diastolic BP than patients with the G/G genotype (A/A: 74.4±9.8, G/A: 78.0±10.8, G/G: 82.4±13.5 mmHg; P = 1×10^−10^). It is worth noting that the exclusion of black patients from all these comparisons did not significantly change the data presented in Table 2.

**Table 2:** Clinical and laboratory characteristics of patients with type 1 diabetes mellitus, broken down by the different genotypes of the *IFIH1* rs1990760 (G/A) polymorphism.

*IFIH1* rs1990760 (G/A) polymorphism				
	G/G (n = 114)	G/A (n = 263)	A/A (n = 150)	P*
Age (years)	36.1±12.6	34.9±14.6	32.8±12.5	0.356
Gender (% male)	51.1	50.5	56.2	0.581
Age of diagnosis (years)	19.2±9.9	16.5±9.7	16.7±11.0	0.298
BMI (kg/m^2^)	22.5±6.5	23.8±4.4	22.2±4.9	0.088
Diabetic nephropathy (%)	35.3	30.3	28.0	0.679
Diabetic retinopathy (%)	50.0	43.7	39.4	0.352
Systolic blood pressure (mm/Hg)†	128.5±18.9^a^	121.1±19.0^b^	117.41±16.7^b^	0.001
Diastolic blood pressure (mm/Hg)†	82.4±13.5^a^	78.0±10.8^b^	74.4±9.8^c^	1×10^−10^
Arterial hypertension (%)†	45.3^a^	33.8^a^	16.1^b^	1×10^−10^
Triglycerides (mmol/L)	0.86 (0.93–1.48)	0.94 (1.08–1.46)	0.84 (0.90–1.98)	0.248
Creatinine (µmol/L)	88.4 (97.2–185.6)	88.4 (88.4–123.7)	79.6 (79.6–114.9)	0.265
HbA1c (% [mmol/mol])	9.0±2.2 [75±0.5]	8.6±2.2 [70±0.5]	9.24±7.0 [77±53]	0.465
HDL cholesterol (mmol/L)	1.5±0.6	1.5±0.4	1.5±0.5	0.664
Total cholesterol (mmol/L)	4.7±1.2	4.8±1.2	4.5±1.1	0.184

Data are expressed as mean ± SD, median (minimum–maximum values), or percentage.

P-values were obtained by One-Way ANOVA or χ^2^ tests, as appropriate.

n = number of subjects. HbA1c = glicohemoglobin.

Only P values lower than the Bonferroni threshold (P = 0.0038) were considered statistically significant.

Different letters (a,b,c) mean that values were significantly different using Tukey’s post hoc test or adjusted-residuals (P<0.05).

To investigate the association between the rs1990760 (G/A) polymorphism and systolic and diastolic BP in greater depth, we further analyzed systolic and diastolic BP levels according to different genetic inheritance models: additive, recessive or dominant [30] (**Table 3**). When taking into account the additive model, both systolic and diastolic BP were lower in A/A genotype carriers as compared to G/G genotype carriers, adjusting for age, gender, ethnicity, presence of DN and treatment for hypertension (systolic BP: Beta = −13.972, P = 0.034, and diastolic BP: Beta = −12.186, P = 0.013). In the same way, when taking into account the dominant model, A allele carriers showed lower levels of both systolic and diastolic BP as compared to G/G genotype carriers, adjusting for age, gender, ethnicity, presence of DN and treatment for hypertension (systolic BP: Beta = −15.814, P = 0.019, and diastolic BP: Beta = −11.771, P = 0.003). Systolic and diastolic BP did not differ statistically among the rs1990760 genotypes under a recessive inheritance model (**Table 3**).

**Table 3:** Systolic and diastolic blood pressure levels in T1DM patients broken down by different inheritance models of the *IFIH1* rs1990760 (G/A) polymorphism.

Models	Systolic BP	Unadjusted P*	Beta/adjusted P†	Diastolic BP	Unadjusted P*	Beta/adjusted P†
Additive model						
G/G	128.5±18.9	1×10^−10^	−14.384/0.034	82.4±13.5	1×10^−10^	−12.525/0.013
A/A	117.4±16.7			74.4±9.8		
Recessive model						
G/A-G/G	123.5±19.2	0.007	−2.271/0.717	79.4±11.9	1×10^−10^	−3.512/0.361
A/A	117.4±16.7			74.4±9.8		
Dominant model						
G/G	128.5±18.9	1×10^−10^	−15.649/0.019	82.4±13.5	1×10^−10^	−12.155/0.003
G/A-A/A	119.7±18.2			76.6±10.6		

Data are expressed as mean ± SD.

P-values were obtained by Student’s t-test.

P values were computed using logistic regression analysis and are adjusted for age, gender, ethnicity, presence of diabetic nephropathy and treatment for hypertension.

Accordingly, the prevalence of AH was lower in T1DM patients carrying the A/A genotype as compared to patients with the G/G genotype (16.1% *vs*. 45.3%; adjusted residuals P<0.010). The association of the A/A genotype with protection to AH was confirmed after adjustment for age, gender, presence of DN and ethnicity (OR = 0.339 for the A/A genotype, P = 0.019). Taking into account that ADA guidelines suggest that the cutoff for the diagnosis of AH in diabetic patients should be BP levels >130/80 mmHg [34], we reanalyzed our data using these values. In agreement with the results described above, the A/A genotype remained significantly associated with protection to AH under an additive model of inheritance and after adjustment for covariates (OR = 0.334, 95% CI = 0.180–0.622, P = 0.003). The prevalence of AH was 52.8% in white subjects and 29.4% in black subjects (P = 0.009). Excluding black subjects from the T1DM sample did not change the above mentioned results regarding the association between the analyzed polymorphism and AH.

In addition, we analyzed 725 patients with T2DM to know better whether the association of the rs1990760 A allele with systolic and diastolic BP was specific to T1DM or could be observed in a non-autoimmune diabetes context. The frequency of the A allele was 0.49 in this sample, and it did not differ from nondiabetic subjects (P = 0.942). Systolic BP did not differ significantly among T2DM patients broken down by the different genotypes of the rs1990760 polymorphism, adjusting for age, sex, ethnicity, BMI and treatment for hypertension (A/A: 145.7±22.7, A/G: 141.5±23.0, G/G; 144.5±24.2 mmHg; P = 0.755). Diastolic BP in these patients also was similar among the three genotypes and controlling for the same covariates (A/A: 85.6±12.9, G/A: 85.3±13.9, G/G: 86.1±14.1 mmHg; P = 0.800) (**Table S1 in File S1**). Accordingly, prevalence of AH also did not differ among different genotypes after adjustment for covariates (P = 0.163; **Table S1 in File S1**).

### *IFIH1* Gene Expression in Mononuclear Cells from a Sub-sample of T1DM Patients

**Figure 2:**
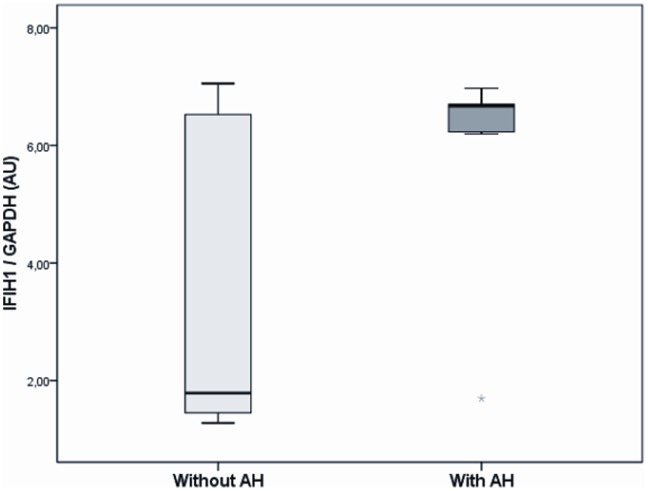
*IFIH1* gene expressions in mononuclear cells from type 1 diabetic patients broken down by the presence of arterial hypertension (AH) (P = 0.036). P values were obtained using Student-s t-test. Data are presented as median (95% CI). AU = arbitrary units.

The median (minimum – maximum values) of *IFIH1* mRNA concentrations in mononuclear cell samples from 26 T1DM patients was 6.3 (1.3–7.1) AU. *IFIH1* gene expression in this sample did not differ significantly among the three genotypes of the rs11990760 polymorphism [G/G (n = 7): 5.3 (1.4–6.8), G/A (n = 13): 6.6 (1.3–7.1), A/A (n = 7): 1.8 (1.3–6.7) AU; P = 0.274]. Nevertheless, *IFIH1* mRNA concentrations were increased in mononuclear cells from T1DM patients with AH (n = 7) as compared with T1DM patients without AH (n = 19) [6.7 (1.7–2.0) *vs*. 1.8 (1.3–7.0 AU), respectively; P = 0.036; **Figure 2**].

### Meta-analysis for the association of the *IFIH1* rs1990760 polymorphism with T1DM

The strategy used to identify and select studies for inclusion in the meta-analysis is shown in **Figure 1**. A total of 7 articles fulfilled the eligibility criteria and were included in the meta-analysis: 6 that had been identified through the database searches [15], [16], [17], [35], [36], [37] in addition to the case-control study we describe above, which was also included in the analysis. NOS scale was used to assess the quality of the studies. In general, most of the studies included in our meta-analysis were considered as having a moderate quality (more than five stars) regarding selection, comparability and exposure. Only one study scored less than four stars.

**Table S2** **in File S1** lists the genotype and allele distributions and OR (95% CI) for the *IFIH1* rs1990760 polymorphism in case and control samples from the different studies reviewed. **Table S3** **in File S1** summarizes the results of the pooled analyses for association between the *IFIH1* polymorphism and susceptibility to T1DM. Our results revealed significant associations between the rs1990760 A allele and risk for T1DM for all analyzed inheritance models: allele contrast (OR = 1.190, 95% CI = 1.160–1.230; **Figure 3**) dominant (OR = 1.260, 95% CI = 1.184–1.342), recessive (OR = 1.236, 95% CI = 1.180–1.294), and additive (OR = 1.404, 95% CI = 1.310–1.505) models. No significant publication bias was detected for any of the inheritance models assessed for the *IFIH1* rs1990760 polymorphism (data not show), which suggests that our data are statistically robust.

**Figure 3:**
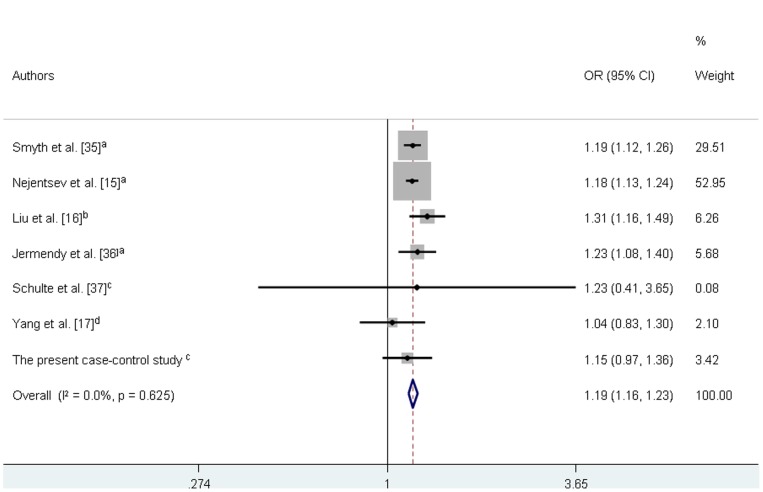
Forest plot showing individual and pooled ORs (95% CI) for the association between the *IFIH1* rs1990760 polymorphism and type 1 diabetes mellitus under an additive inheritance model. The areas of the squares reflect the weight of each individual study and the diamond illustrates the fixed-effect model summary OR (95% CI). ^a^ = Europeans,^ b^ = Caucasian, ^c^ = Asian, ^d = ^mixed population.

## Discussion

In response to viral infections, the CARD domain of IFIH1 receptor associate with the CARD-containing adaptor molecule known as IPS-1 (IFN promoter stimulator-1), activating the transcription factors NF-κB and IRF-3, which then cooperate in induction of antiviral IFN-I response [12], [13], [38]. This local inflammation coupled with triggering of antiviral defenses will in most cases eradicate the viral infection. However, in some genetically susceptible subjects, these cellular attempts to eradicate the infection might go wrong; thus, predisposing for T1DM developing [11].

An association between the *IFIH1* gene and T1DM was first reported by Smyth *et al*. [35], who performed a genome-wide association scan in European families with T1DM. Several polymorphisms located within the *IFIH1* gene region showed an association with T1DM, with the rs1990760 polymorphism being the most strongly associated with the disease (OR = 0.86, P = 1.4×10^−10^ for the G allele). This association between the rs1990760 polymorphism and T1DM was replicated in other populations [15], [16], [36], [39], and a recent meta-analysis provided additional evidence for an influence of the A allele on T1DM risk in Europeans [40]. In the present study, we were able to replicate the reported association between the rs1990760 A allele and risk for T1DM with a case-control study followed by a meta-analysis of the literature on the subject.

The exact mechanisms by which *IFIH1* polymorphisms contribute to T1DM pathogenesis remain to be explored. The rs1990760 polymorphism is not located in any functional region of the IFIH1 receptor, but the G allele is highly conserved among mammals and may have other, yet-unknown functions or may affect active domains through effects on the tertiary structure. Liu *et al*. [16] reported that the G/G genotype of this polymorphism was associated with 1.2–2.0 fold-increase in *IFIH1* gene expression in mononuclear cells of 374 subjects. Although our present data showed that *IFIH1* expression is 3.5 fold higher in mononuclear cells from T1DM patients carrying the G/G genotype when compared with A/A genotype patients, this difference did not reach formal statistical significance probably due to the small sample size analyzed. McCartney *et al*. [41] reported that optimal functioning of IFIH1 and prompt IFN-I response are required to prevent diabetes in C57BL/6 mice infected with encephalomyocarditis virus strain D, which has tropism for pancreatic beta-cells. Taking these data into consideration, we therefore hypothesize that probably due to a lower *IFIH1* expression, A/A genotype carriers might have a suboptimal IFIH1 function and, consequently, might be more predisposed to develop T1DM.

Our present results showed that age, age at T1DM diagnosis, proportion of males, BMI, creatinine levels, lipid profile, HbA1c and presence of DR or DN did not differ among the three genotypes of the rs1990760 polymorphism. Surprisingly, we observed that T1DM patients carrying the A allele had lower levels of both systolic and diastolic BP as compared to patients with the G/G genotype under additive and dominant models of inheritance. The A/A genotype seems to be associated with significant protection for AH. To our knowledge, this is the first time that an *IFIH1* polymorphism is reported as being probably associated with AH.

Hypertension is a common disorder with uncertain etiology [42]. However, in the last years, it has become evident that components of both the innate and adaptive immune systems play an important role in hypertension [42], [43], [44]. For example, in mice lacking vascular macrophages, angiotensin-II and deoxycorticosterone acetate-salt treatment are unable to raise BP [45], [46], corroborating the role of innate immunity in hypertension. Therefore, taking into account the role of low-grade inflammation in the pathogenesis of hypertension [42], [44], [47], [48], [49], our present results suggesting an association between the rs1990760 A allele and protection for AH seem to be biologically plausible albeit not yet completely understood.

Recently, Chatterjee *et al*. [50], [51] demonstrated that all the three dsRNAs receptors, TLR-3, RIG-I and IFIH1, are activated in placentas of mice and women with preeclampsia (PE), a pregnancy-specific syndrome characterized by excessive maternal immune system activation, inflammation, and endothelial dysfunction, causing hypertension. Chatterjee *et al*. [50] also showed that treatment of mice with intracellular synthetic dsRNA (PIC) significantly increased TLR3 levels and caused pregnancy-dependent hypertension, endothelial dysfunction and placental inflammation. Our results suggesting that *IFIH1* gene expression is increased in mononuclear cell from T1DM patients with AH is in agreement with the data of Chatterjee *et al*. [50], [51] indicating that high expression of dsRNA receptors may have a role in hypertension. Therefore, it seems reasonable to suggest that T1DM patients carrying the A/A genotype may have a lower risk for developing AH due to a decreased *IFIH1* gene expression and, consequently, a lesser inflammatory environment.

Our data indicating that the *IFIH1* rs1990760 polymorphism is possibly associated with AH in T1DM patients but not in patients with T2DM may be suggestive that the role of this gene in hypertension is dependent of an immune attack as occurring in the development of T1DM or PE. Another possibility is that in T2DM, AH would be more influenced by age, obesity, and insulin resistance than by genetic polymorphism related to inflammation. Consequently, in this group of patients, the effect of the rs1990760 polymorphism in AH would be less important than in the T1DM population. Further studies are needed to confirm the association of the rs1990760 polymorphism with protection for AH in other populations and to evaluate which ligand activates the IFIH1 receptor and how increased levels of this dsRNA receptor lead to AH.

In conclusion, our results confirm the association between the A allele of the *IFIH1* rs1990760 polymorphism and T1DM in our population and in a meta-analysis of the literature on the subject. Moreover, the A allele seems to be associated with a protection for AH in T1DM patients. This study adds a new possible role of IFIH1 in the pathogenesis of AH in T1DM patients.

## Supporting Information

File S1 Supporting tables. Table S1, Clinical and laboratory characteristics of patients with type 2 diabetes mellitus, broken down by the different genotypes of the *IFIH1* rs1990760 (G/A) polymorphism. Table S2, Genotype and allele distributions of the *IFIH1* rs1990760 polymorphism in patients with type 1 diabetes mellitus (cases) and nondiabetic subjects (controls) for each individual study included in the meta-analysis. Table S3, Pooled measures for associations between the *IFIH1* rs1990760 polymorphism and susceptibility for type 1 diabetes mellitus, under different inheritance models.
(DOC)
