# Supplementary material for: The A Allele of the rs1990760 Polymorphism in the IFIH1 Gene Is Associated with Protection for Arterial Hypertension in Type 1 Diabetic Patients and with Expression of This Gene in Human Mononuclear Cells
Source: PLoS One. 2013 Dec 27;8(12):e83451. doi: 10.1371/journal.pone.0083451 (PMC3873949; doi:10.1371/journal.pone.0083451)
Supplement: File S1 — Supporting tables. Table S1, Clinical and laboratory characteristics of patients with type 2 diabetes mellitus, broken down by the different genotypes of the IFIH1 rs1990760 (G/A) polymorphism. Table S2, Genotype and allele distributions of the IFIH1 rs1990760 polymorphism in patients with type 1 diabetes mellitus (cases) and nondiabetic subjects (controls) for each individual study included in the meta-analysis. Table S3, Pooled measures for associations between the IFIH1 rs1990760 polymorphism and susceptibility for type 1 diabetes mellitus, under different inheritance models. (DOC) [file pone.0083451.s001.doc]

**Table S1.** Clinical and laboratory characteristics of patients with type 2 diabetes mellitus, broken down by the different genotypes of the *IFIH1* rs1990760 (G/A) polymorphism.

| **rs1990760 (G/A) polymorphism** | | | | | |
| --- | --- | --- | --- | --- | --- |
|  | **Total sample** | **G/G (n = 266)** | **G/A (n = 301)** | **A/A (n = 269)** | P* |
| Age (years) | 55.0 ± 11.5 | 55,0 ± 11.6 | 54.2 ± 11.1 | 56.3 ± 11.8 | 0.055 |
| Gender (% male) | 50.4 | 46.1 | 51.3 | 53.4 | 0.199 |
| Age of diagnosis (years) | 46.4 ± 10.8 | 45.6 ± 11.5 | 46.2 ± 10.2 | 47.4 ± 10.7 | 0.256 |
| BMI (kg/m^2^) | 28.9 ± 5.3 | 28.9 ± 5.2 | 28.6 ± 5.1 | 29.4 ± 5.6 | 0.209 |
| Diabetic nephropathy (%) | 48.4 | 51.4 | 46.0 | 48.8 | 0.405 |
| Diabetic retinopathy (%) | 52.0 | 54.5 | 47.6 | 55.4 | 0.223 |
| Systolic blood pressure (mm/Hg) | 144.5 ± 24.2 | 144.5 ± 24.2 | 141.5 ± 23.0 | 145.7 ± 22.7 | 0.755 |
| Diastolic blood pressure (mm/Hg) | 86.1 ± 14.1 | 86.1 ± 14.1 | 85.3 ± 13.9 | 85.6 ± 12.9 | 0.800 |
| Triglycerides (mmol/L) | 1.69 (0.30-13.95) | 1.60 (0.51-13.95) | 0.53 (0.53-8.43) | 1.78 (0.30-9.3) | 0.127 |
| Creatinine (µmol/L) | 79.5 (35.3-1193.4) | 79.5 (35.3-945.8) | 79.5 (35.3-1193.4) | 79.5 (35.3-937.0) | 0.633 |
| HbA1c (%) | 7.7 ± 1.7 | 7.8 ± 1.8 | 7.6 ± 1.7 | 7.9 ± 1.8 | 0.492 |
| HDL cholesterol (mmol/L) | 1.1 ± 0.3 | 1.2 ± 0.3 | 1.1 ± 0.3 | 1.1 ± 0.4 | 0.103 |
| Total cholesterol (mmol/L) | 5.2 ± 1.1 | 5.2 ± 1.1 | 5.2 ± 1.1 | 5.3 ± 1.1 | 0.870 |

Data are expressed as mean ± SD, median (minimum–maximum values), or percentage.

*P-values were obtained by One-Way ANOVA or χ^2^ tests, as appropriate.

n = number of subjects. HbA1c = glicohemoglobin.

**Table S2.** Genotype and allele distributions of the rs1990760 (G/A) polymorphism in patients with type 1 diabetes mellitus (cases) and non-diabetic subjects (controls)

| **rs1990760 (G/A)** | | | **Cases (n) by total and genotype** | | | | **Controls (n) by total and genotype** | | | | **A allele frequency (%)** | | |
| --- | --- | --- | --- | --- | --- | --- | --- | --- | --- | --- | --- | --- | --- |
| Reference | Year | Ethnicity | Total | A/A | G/A | G/G | Total | A/A | G/A | G/G | Cases | Controls | OR (95% CI) |
| Smyth *et al.* | 2006 | Europeans | 4253 | 1810 | 1906 | 537 | 5842 | 2183 | 2751 | 908 | 0.64 | 0.60 | 1.189 (1.228 – 1.261) |
| Nejentsev *et al.* | 2009 | Europeans | 7759 | 3280 | 3502 | 977 | 10175 | 3789 | 4813 | 1573 | 0.64 | 0.60 | 1.184 (1.134 – 1.237) |
| Jermendy *et al*. | 2010 | Europeans | 1266 | 516 | 566 | 184 | 749 | 266 | 340 | 143 | 0.63 | 0.57 | 1.222 (1.077 – 1.399) |
| Liu *et al.* | 2009 | Caucasian | 2046 | - | - | - | 2417 | - | - | - | 0.67 | 0.60 | 1.310 (1.161 – 1.485) |
| Schulte *et al.* | 2010 | Mixed | 10 | 3 | 6 | 1 | 20 | 5 | 12 | 3 | 0.60 | 0.68 | 1.222 (0.4126 – 1.365) |
| Yang *et al.* | 2011 | Asian | 464 | 27 | 140 | 297 | 465 | 15 | 159 | 291 | 0.20 | 0.20 | 1.036 (0.827 – 1.297) |
| The present case-control study | 2013 | Mixed | 527 | 150 | 263 | 114 | 517 | 139 | 239 | 139 | 0.53 | 0.50 | 1.146 (0.965 – 1.361) |

**Table S3. Pooled measures for associations between the rs1990760 G/A polymorphism and susceptibility to T1DM.**

| **Inheritance model** | **n studies** | **n cases** | **n controls** | **I² (%)** | **Pooled OR (95% CI)** |
| --- | --- | --- | --- | --- | --- |
| **rs1990760** |  |  |  |  |  |
| Allele contrast | 7 | 30604 | 37953 | 0.0 | 1.190 (1.160 - 1.230) |
| Additive | 6 | 7896 | 9454 | 0.0 | 1.404 (1.310 - 1.505) |
| Dominant | 6 | 14279 | 17768 | 8.8 | 1.260 (1.184 -1.342) |
| Recessive | 6 | 14279 | 17768 | 0.0 | 1.236 (1.180 -1.294) |

The fixed effect model (FEM) was used for the calculation of the pooled OR (95% CI).
